# Eruption Disturbance in Children Receiving Bisphosphonates: Two Case Reports

**DOI:** 10.3390/ph17111521

**Published:** 2024-11-12

**Authors:** Tatsuya Akitomo, Yuko Iwamoto, Mariko Kametani, Ami Kaneki, Taku Nishimura, Chieko Mitsuhata, Ryota Nomura

**Affiliations:** Department of Pediatric Dentistry, Graduate School of Biomedical and Health Sciences, Hiroshima University, 1-2-3 Kasumi, Minami-ku, Hiroshima 734-8553, Japan; yuko-tulip@hiroshima-u.ac.jp (Y.I.); mrysk25@hiroshima-u.ac.jp (M.K.); kaneki@hiroshima-u.ac.jp (A.K.); nishi04@hiroshima-u.ac.jp (T.N.); chiekom@hiroshima-u.ac.jp (C.M.); rnomura@hiroshima-u.ac.jp (R.N.)

**Keywords:** eruption disturbance, bisphosphonates, pediatric dentistry, case report

## Abstract

Background: Bisphosphonates used for the treatment of osteoporosis, hypercalcemia, or heterotopic calcifications can cause serious adverse dental events such as osteonecrosis of the maxillary and mandibular bones. However, the effects in childhood remain scarcely explored. Case Presentations: We encountered two children who had started bisphosphonate therapy before completion of the primary dentition. No systemic disease causing congenital delayed tooth eruption was diagnosed. Although the children’s height and weight increased with age, their tooth eruption was significantly delayed compared with the mean. The primary teeth gradually erupted in the follow-up period; however, some teeth did not completely erupt and needed to be extracted to allow for permanent tooth eruption. Conclusions: We report a case of children with early use of bisphosphonates and eruption disturbance, highlighting the need for further investigation into the relationship between these factors.

## 1. Introduction

When alendronate received regulatory approval for the treatment of postmenopausal osteoporosis in 1995, bisphosphonates were prescribed for a range of additional indications, including Paget’s disease of bone, prevention of skeletal-related events in advanced malignancy, hypercalcemia of malignancy, and osteoporosis in men [1]. Although bisphosphonates have huge clinical benefits, various adverse reactions such as renal failure and flulike symptoms with fever have been reported. Additionally, gastrointestinal disorders such as dyspepsia and esophagitis may occur after oral administration [2]. In the dental field, bisphosphonates are associated with a serious adverse event: osteonecrosis of the jaw. This condition is called medication-related osteonecrosis of the jaw (MRONJ), and it is among the most severe side effects of bisphosphonate therapy [2,3].

In recent years, bisphosphonates have also been used in children for treatment of a growing number of disorders associated with osteoporosis, resistant hypercalcemia, or heterotopic calcifications [4]. A recent position paper stated that there have been no reports of MRONJ development in bisphosphonate-treated children with osteogenesis imperfecta [5]. However, Malmgren et al. (2021) reported that starting bisphosphonate therapy before the age of 2 years increases the risk of abnormalities in tooth formation, manifesting as morphological aberrations, tooth agenesis, and enamel defects [6]. Baroncelli et al. (2014) concluded that there is insufficient evidence for the routine use of bisphosphonates in clinical care and that their use in children should be carefully weighed to ensure that the potential benefits to bone health outweigh any potential risks [4].

As mentioned above, the oral side effects of bisphosphonates in children are scarcely explored. However, it has been reported that the administration of zoledronic acid, a type of bisphosphonate, to newborn mice irreversibly inhibited tooth eruption and elongation [7]. Here, we describe two cases of children who received bisphosphonate therapy before the age of 2 years, both of whom exhibited eruption disturbances of the primary teeth. This report describes the oral condition of the two patients and reviews their clinical characteristics.

## 2. Detailed Case Description

This study followed the CARE guidelines, which are provided via the Internet (https://static1.squarespace.com/static/5db7b349364ff063a6c58ab8/t/5db7bf175f869e5812fd4293/1572323098501/CARE-checklist-English-2013.pdf (accessed on 8 November 2024)) (Supplementary Table S1).

### 2.1. Case 1

A boy aged 1 year and 8 months old was referred from the pediatric department of our hospital with a chief complaint of dental caries. His primary first molars had already erupted, and 16 primary teeth were present in the oral cavity. Dental caries was observed in the maxillary right primary canine. He had received bisphosphonate treatment (intravenous zoledronic acid, 1 mg/month) since the age of 1 year and 6 months for treatment of Langerhans cell histiocytosis.

**Figure 1:**
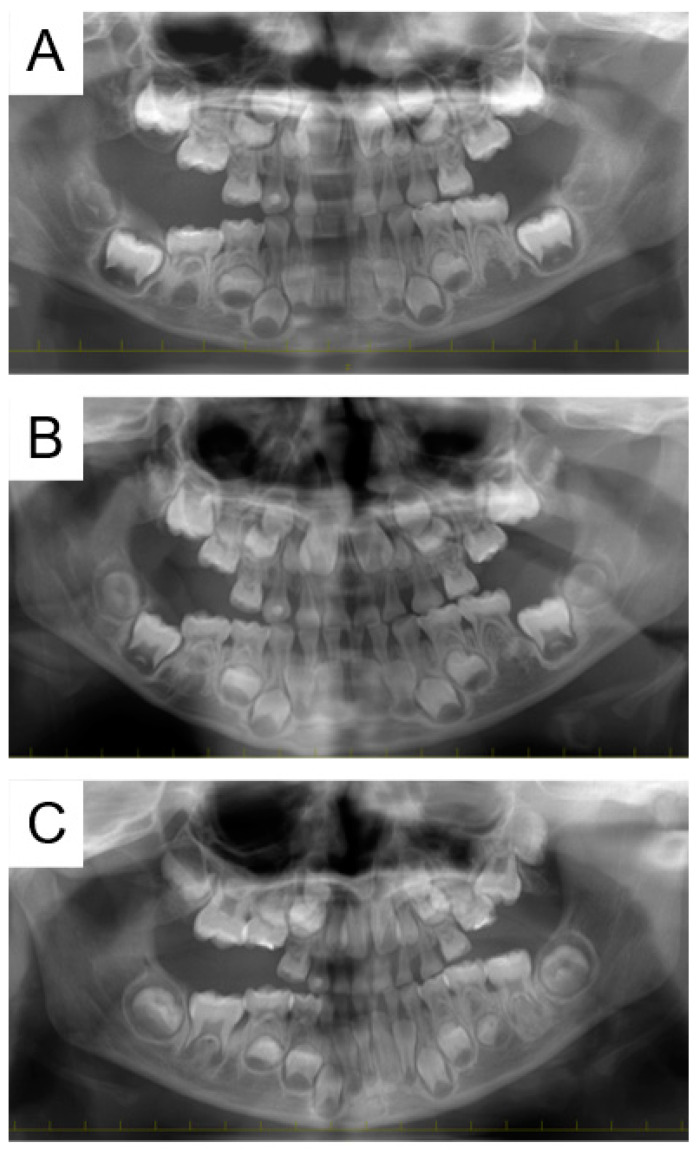
Panoramic radiographs of Case 1 revealing the diagnosis as delayed eruption of the maxillary primary second molars, and the location was not changed despite traction treatment. Images taken at (**A**) 4 years and 6 months, (**B**) 5 years and 3 months, and (**C**) 6 years and 6 months.

We treated the dental caries and continued oral management. At the age of 2 years and 5 months, the mandibular left primary second molar started to erupt, and the mandibular right primary second molar erupted at 2 years and 11 months. Bisphosphonate treatment was completed afterwards at the age of 3 years, and the maxillary left primary second molar was detected 2 months later. At 3 years and 7 months, the maxillary right primary second molar started to erupt. However, three of the primary molars (except the mandibular left primary second molar) did not reach the height of the occlusion, and panoramic radiographs taken at 4 years and 6 months confirmed delayed eruption (Figure 1A). Reevaluation 9 months later confirmed eruption of the mandibular right second molar; however, there was no change in the maxillary second molars (Figure 1B).

**Figure 2:**
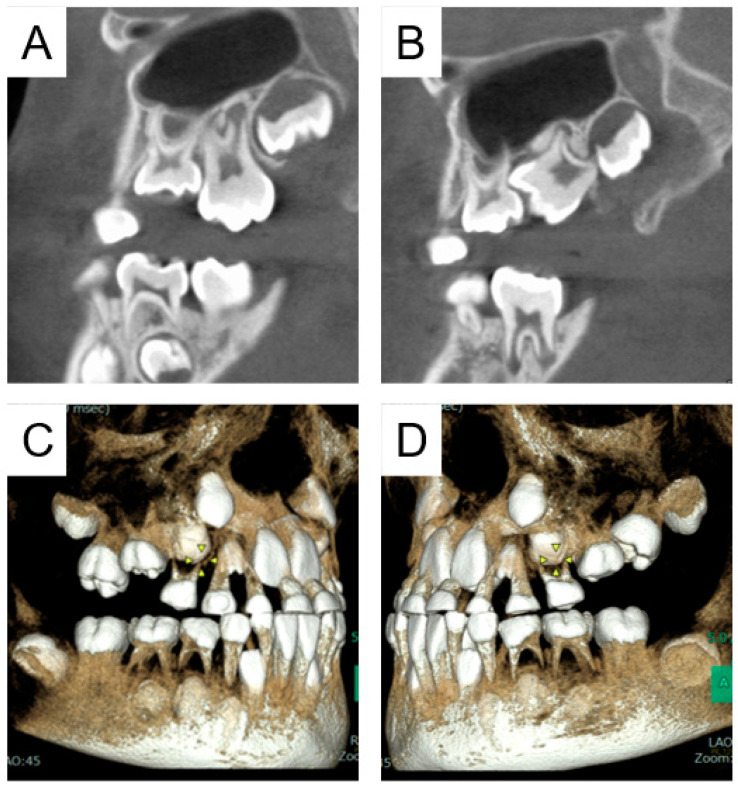
Cone-beam computed tomography images of Case 1 obtained at 7 years 0 months showing eruption disturbance of the maxillary left permanent first molar. Sagittal section at maxillary right first molar (**A**) and left first molar (**B**). Three-dimensional construction images at right (**C**) and left (**D**).

**Figure 3:**
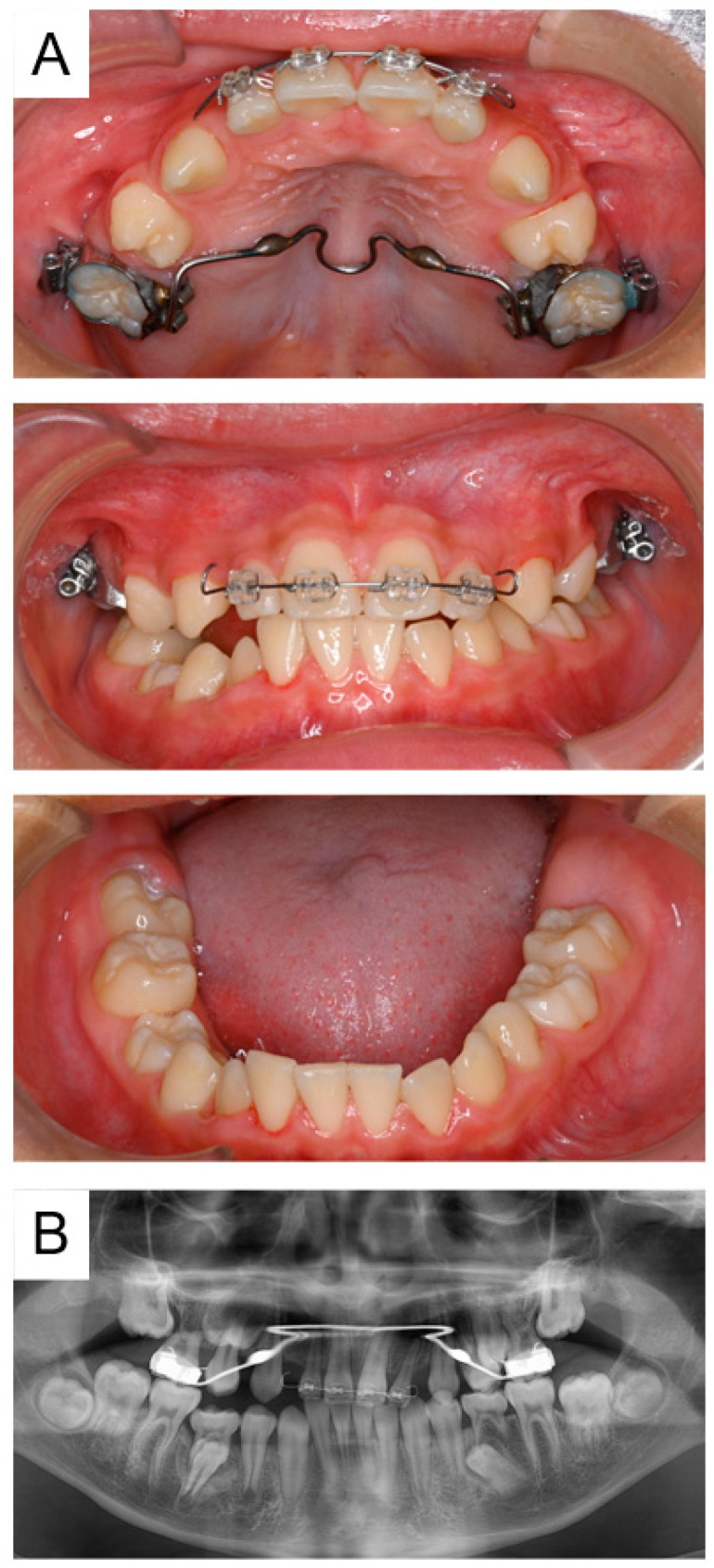
Images of Case 1 at the age of 12 years and 5 months showing the ankylosis of the mandibular right primary second molar. Intraoral photographs (**A**) and panoramic radiograph (**B**).

We started traction treatment of the maxillary left primary second molar at 5 years and 8 months; however, the location of the tooth did not change. Panoramic radiographs indicated eruption disturbance of the maxillary left permanent first molar (Figure 1C). Cone-beam computed tomography images were taken at 7 years for more detailed information (Figure 2), and the bilateral maxillary primary second molars were extracted in the oral surgery department under general anesthesia. Five months later, eruption of the maxillary left permanent first molar was confirmed. At the age of 12 years and 5 months, panoramic radiographs revealed ankylosis of the mandibular right primary second molar and an abnormal eruption direction of the mandibular second premolars (Figure 3). Orthodontic treatment was continued by an orthodontic specialist, and no obvious eruption disturbance of the permanent teeth was detected. We will continue to monitor the progress of the patient, along with pediatric consultations.

### 2.2. Case 2

A 14-month-old boy referred from the pediatric department of our hospital presented with delayed tooth eruption. He was born at 36 weeks gestation with a height of 44 cm and weight of 2531 g. Soon after birth, he was diagnosed with decreased muscle tone, respiratory problems, joint contractures, and suspected myopathy. In myopathy, abnormalities in the bones can sometimes be observed. Upon diagnosis, the patient was found to have osteoporosis based on bone mineral density, and medication with bisphosphonates (oral alendronic acid, 5 mg/week) was started at the age of 3 months. The dosage of bisphosphonates was changed with growth to 7 mg/week two weeks later, 8.75 mg/week at the age of 4 months, 10.5 mg/week at the 6 months, and 14 mg/week at the 1 years.

**Figure 4:**
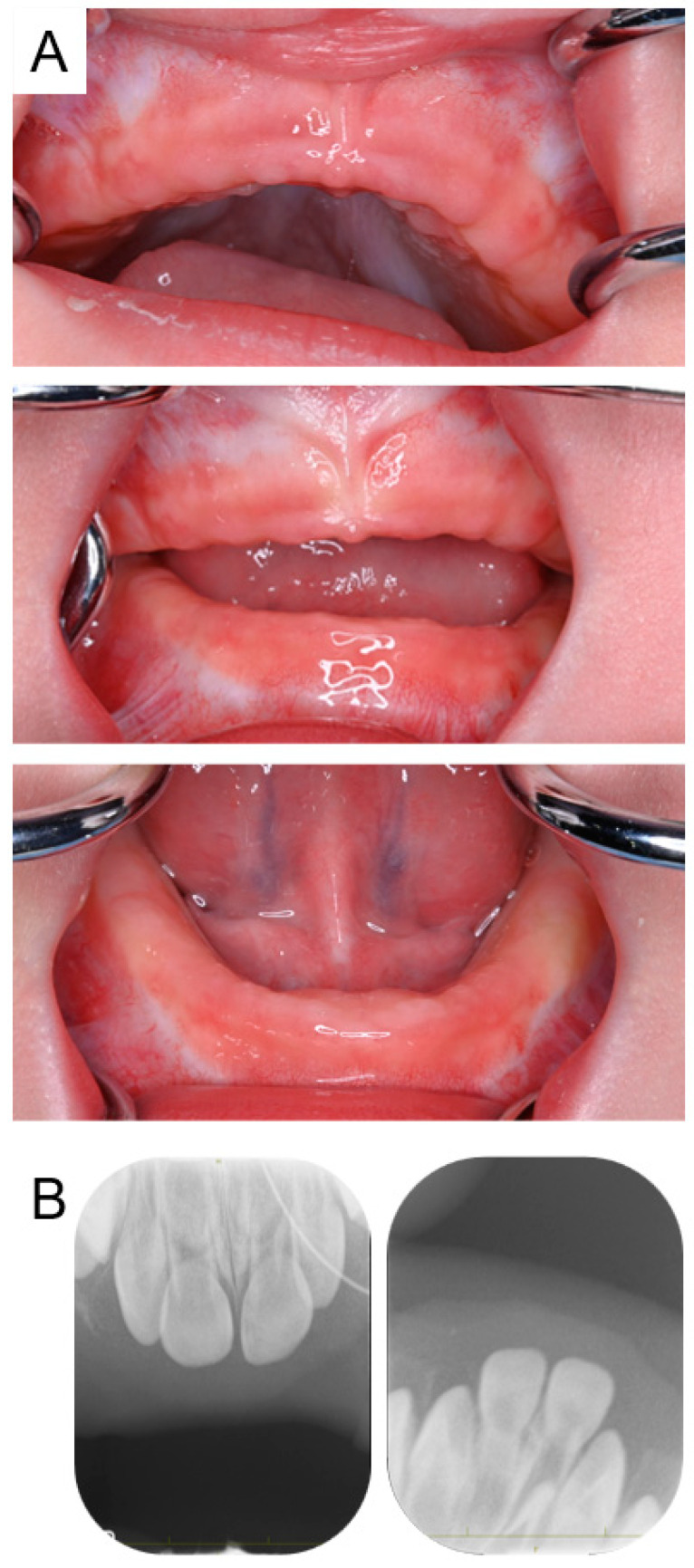
Images of Case 2 at the age of 1 year and 2 months showing suspected delayed eruption of primary incisors. Intraoral photographs (**A**) and periapical radiographs (**B**).

**Figure 5:**
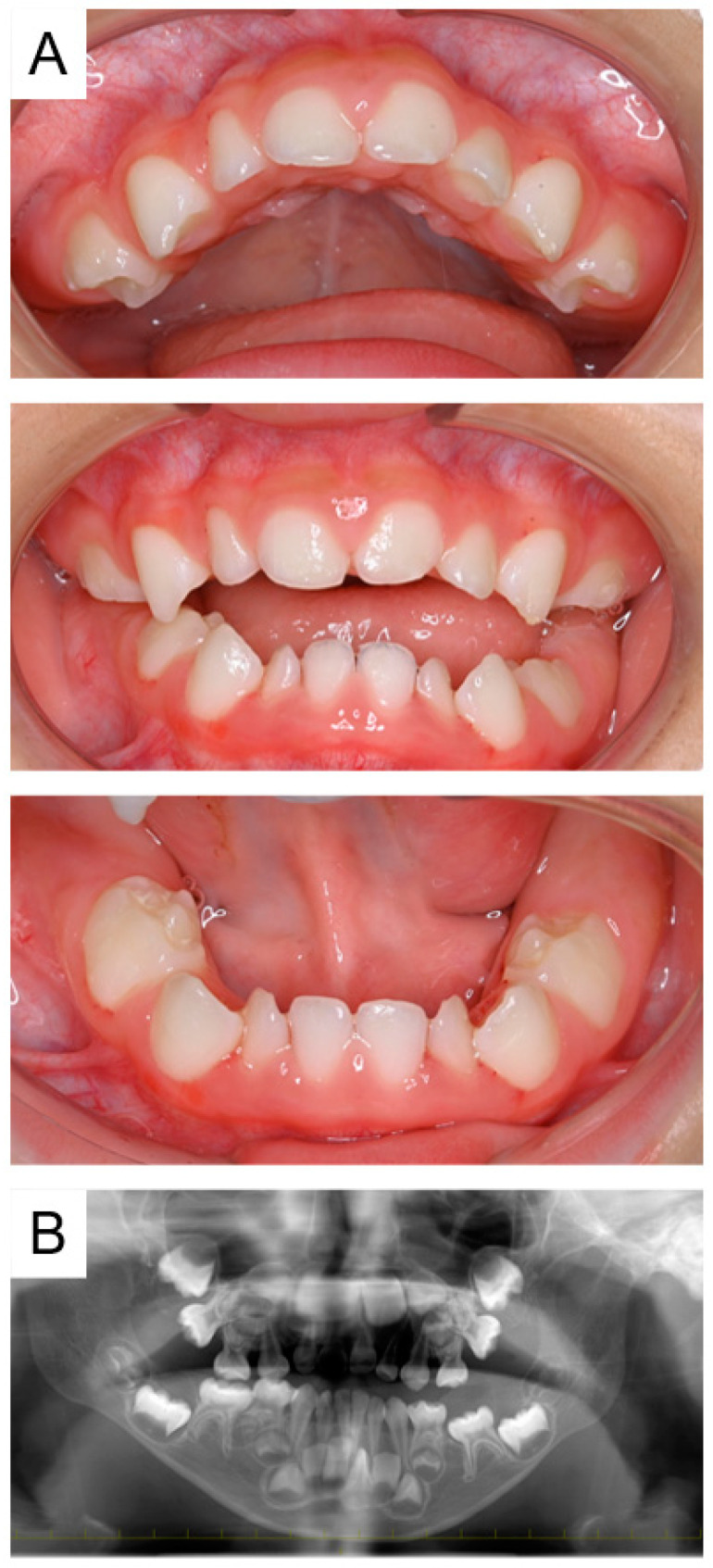
Images of Case 2 at the age of 4 years and 1 month revealing eruption disturbance of the primary second molars. Intraoral photographs (**A**) and panoramic radiograph (**B**).

Intraoral examination revealed that there were no primary teeth in the oral cavity; however, primary incisors were visible on periapical radiographs (Figure 4). At the age of 1 year and 8 months, three primary central incisors (except the mandibular right incisor) had erupted, and the mandibular right incisor also erupted 2 months later. Bisphosphonate treatment was completed at the age of 2 years, and the patient was diagnosed with neurogenic arthrogryposis multiplex by muscle biopsy at 3 years and 2 months. At the age of 4 years and 1 month, all primary first molars had erupted; however, panoramic radiographs indicated eruption disturbance of the primary second molars (Figure 5). Three primary second molars had not erupted at the age of 4 years and 5 months, and we are continuing oral management at follow-up appointments.

## 3. Discussion

In this report, we described the progress of children who started taking bisphosphonate preparations before the completion of the primary dentition. The patients’ clinical information is summarized in Table 1. Patients with osteogenesis imperfecta, a target of bisphosphonate therapy, are reported to experience malocclusion, hypodontia, and impacted teeth [8,9]. The impacted teeth may be congenitally associated with the systemic disease. However, in the two cases discussed, the systemic diseases were Langerhans cell histiocytosis and neurogenic arthrogryposis multiplex. Although some reports have described oral manifestations of Langerhans cell histiocytosis, none have involved impacted teeth [10,11,12].

The most common maxillofacial symptoms in arthrogryposis multiplex congenita are mandibular hypoplasia, limited mandibular opening, and high-arched palate [13]. Taqi et al. (2024) published a review of dental anomalies in individuals with arthrogryposis multiplex congenita and reported delayed eruption and enamel hypoplasia in patients who also had ectodermal dysplasia [14,15]. Tuffli et al. (1983) also observed that delayed eruption occurred in patients with ectodermal dysplasia, and these reports suggest that the delayed eruption could be associated with ectodermal dysplasia rather than arthrogryposis multiplex congenita [16]. Thus, neither of the two systemic diseases in the present cases is associated with congenital delayed tooth eruption or impacted teeth, suggesting that acquired factors may be involved. To our knowledge, this is the first report describing eruption disturbances in patients receiving bisphosphonate therapy.

The eruption times of the two cases were compared to the mean of Japanese males [17]. In Case 1, bisphosphonates were started at 18 months of age, and 16 erupted teeth were detected at the first visit at 20 months of age. The average age for eruption of 16 primary teeth up to the primary first molar for Japanese males is 17 months; therefore, tooth eruption was not delayed for the patient in Case 1 at that time. However, eruption of the three primary second molars (except the mandibular left primary second molar) was more than 1 S.D. slower than the mean for Japanese males. In Case 2, bisphosphonate treatment started at the age of 3 months, which is earlier than the mean eruption time of all primary teeth. All of the patient’s teeth erupted at least 3 S.D. later than the mean, and three of the primary second molars have not yet erupted. A tooth should be considered a delayed eruption when the emergence of the tooth is more than 2 S.D. from the means of established norms for eruption times [18,19]. According to this definition, the right second primary molars on both the maxilla and mandibula are diagnosed as delayed eruption in Case 1. In addition, it applies to all primary teeth that have erupted to date in Case 2, and all of them were primary teeth that had erupted after bisphosphonate therapy. Eruption times were recorded at intraoral checkups at regular dental visits, so the eruption times are not precisely accurate. However, these results suggest that the eruption of primary teeth may have been delayed after bisphosphonate therapy had begun.

We investigated the growth changes in comparison with the mean for Japanese males [20]. In Case 1, the patient’s height at 1 year was 1 S.D. lower than the mean; however, there was no difference subsequently. The height of the patient in Case 2 was no different than the mean, while the weight at 3 and 4 years was more than 1 S.D. lower. Although lower values for growth were observed at some points in both Case 1 and Case 2, there was no association with the timing of the delayed tooth eruption. Additionally, neither height nor weight showed persistently low values, suggesting that the delayed tooth eruption was not due to growth retardation.

Bisphosphonates are chemically stable derivatives of inorganic pyrophosphate, a naturally occurring compound in which two phosphate groups are linked by esterification [21]. They bind to areas of exposed calcium in the skeleton and cause osteoclast apoptosis, leading to a reduction in remodeling rates, and they have been used as therapeutic agents for osteoporosis and osteopenia [22]. It has also been shown that they inhibit bone resorption by osteoclasts, which suppresses the formation of cancer cell colonies in bone associated with cancers such as breast cancer and multiple myeloma [23]. It is considered that tooth eruption is critically dependent upon the presence of osteoclasts to create an eruption pathway through the alveolar bone. Additionally, bisphosphonates are preferentially incorporated into sites of active bone remodeling [21,24]. There are some reports of dental abnormalities in children who have undergone chemotherapy, and the type of teeth affected depends on the time of commencement [25,26,27]. Therefore, medication may affect the oral cavity, including the developing teeth.

Tooth eruption is executed through three defined stages: pre-eruptive tooth movement, eruptive tooth movement, and post-eruptive tooth movement. Osteoclast activity is particularly important during the second stage of tooth eruption, during which the tooth emerges into the oral cavity [28]. In this stage, the root is formed by the Hertwig epithelial root sheath and the dental mesenchyme, and osteoclasts resorb the bone of the cortical shell on the coronal portion of the tooth bud [28]. Xin et al. (2020) reported that the delayed eruption observed in cleidocranial dysplasia, an autosomal dominant skeletal disorder characterized by impaction of permanent teeth, was caused by dysfunction of osteoclasts [29]. Craniometaphyseal dysplasia, which is associated with prolonged retention of deciduous teeth and delayed eruption of permanent teeth, is also caused by osteoclast dysfunction [30]. The inhibition of osteoclasts by bisphosphonates suppresses bone resorption around the tooth crown, which may result in delayed tooth eruption. Although tooth eruption was delayed in both cases, all the primary second molars were able to erupt in Case 1. The eruption of Case 2 is also being monitored.

Teeth can reach occlusal height eventually even if there is a dental anomaly and eruption is delayed [31,32]. Hayashibara et al. (2000) reported that, although the eruption of permanent teeth may be delayed in craniometaphyseal dysplasia patients, most permanent teeth do erupt naturally [30]. However, the maxillary second molars did not reach occlusal height, and traction treatment could not change their position in Case 1. Primary failure of eruption (PFE) is a rare disorder defined as incomplete tooth eruption despite the presence of a clear eruption pathway [33]. The PTH1R gene is involved in bone metabolism regulation and calcium homeostasis, and various putative loss-of-function mutations in PTH1R are present in patients with PFE [33,34]. However, Jansen’s metaphyseal chondrodysplasia is caused by activation of mutations in PTH1R, leading to hypercalcemia, and bisphosphonates are used to treat this condition [35]. Bisphosphonates induce a state of PTH1R inhibition and may lead to conditions such as PFE. Further investigation of the association between bisphosphonates and PTH1R is required.

This study had some limitations. First, the second primary molars in Case 2 had not all erupted, and the patient’s final outcome was not available. Regular dental checkups enable early detection and treatment. Additionally, it can not only prevent the occurrence of oral diseases but also lead to improvement [36,37]. We will continue the follow-up of these patients until the permanent dentition is complete. Second, in Case 2, the bisphosphonate therapy was completed before eruption time of the primary second molar, and the association is unclear. However, in patients who received chemotherapy, the younger the age at diagnosis or treatment, the higher the prevalence of dental abnormalities [38]. Therefore, bisphosphonate therapy before the tooth eruption time may affect tooth eruption. A third limitation was the small sample size. Only a small number of patients who used bisphosphonates before complete eruption of the primary teeth did not have congenital bone disease. Additional large-scale studies are needed in the future.

## 4. Conclusions

We encountered two patients who had received bisphosphonates before complete eruption of the primary teeth, and both experienced delayed eruption. Additionally, some teeth never completely erupted. This study suggests that eruption disturbances occurred in patients who received bisphosphonate treatment during childhood, indicating the need to further investigate these associations.

## Figures and Tables

**Table 1:** Patients’ clinical information.

	Case 1	Case 2
Age at initial visit	1 y 8 m	1 y 2 m
Diagnosis of systemic disease	Langerhans cell histiocytosis	Neurogenic arthrogryposis multiplex
Type of bisphosphonate	Zoledronic acid	Alendronic acid
Age at start of bisphosphonate treatment	1 y 6 m	3 m
Age at completion of bisphosphonate treatment	3 y 0 m	2 y 0 m
Age at current visit	12 y 5 m	4 y 9 m

## Data Availability

Data are contained within the article and Supplementary Materials.

## Supplementary Materials

The following supporting information can be downloaded at: https://www.mdpi.com/article/10.3390/ph17111521/s1, Supplementary Table S1: CARE Checklist of information to include when writing a case report.
