# Supplementary material for: Eruption Disturbance in Children Receiving Bisphosphonates: Two Case Reports
Source: Pharmaceuticals (Basel). 2024 Nov 12;17(11):1521. doi: 10.3390/ph17111521 (PMC11597784; doi:10.3390/ph17111521)
Supplement: Supplementary file 1 [file pharmaceuticals-17-01521-s001.zip › pharmaceuticals-3290610-supplementary.pdf]

Table S1 CARE Checklist of information to include when writing a case report.

CARE case report guidelines

CARE Checklist of information to include when writing a case report

(cc) BY-NC-ND

Topic	Item	Checklist item description	Reported on Line
Title	1	The diagnosis or intervention of primary focus followed by the words "case report"	Lines 2-3
Key Words	2	2 to 5 key words that identify diagnoses or interventions in this case report, including "case report"	Lines 23
Abstract (no references)	3a	Introduction: What is unique about this case and what does it add to the scientific literature?	Lines 14
	3b	Main symptoms and/or important clinical findings	Lines 15-17
	3c	The main diagnoses, therapeutic interventions, and outcomes	Lines 17-20
	3d	Conclusion—What is the main "take-away" lesson(s) from this case?	Lines 20-22
Introduction	4	One or two paragraphs summarizing why this case is unique (**may include references**)	Lines 46-50
Patient Information	5a	De-identified patient specific information.	Lines 60-61, 98-99
	5b	Primary concerns and symptoms of the patient.	Lines 60-61, 98-99
	5c	Medical, family, and psycho-social history including relevant genetic information	Lines 63-65, 99-106
	5d	Relevant past interventions with outcomes	N / A
Clinical Findings	6	Describe significant physical examination (PE) and important clinical findings.	Lines 61-63, 110-111
Timeline	7	Historical and current information from this episode of care organized as a timeline	N / A
Diagnostic Assessment	8a	Diagnostic testing (such as PE, laboratory testing, imaging, surveys).	Lines 71-74, 115-117
	8b	Diagnostic challenges (such as access to testing, financial, or cultural)	N / A
	8c	Diagnosis (including other diagnoses considered)	Lines 74-75, 115-117
	8d	Prognosis (such as staging in oncology) where applicable	N / A
Therapeutic Intervention	9a	Types of therapeutic intervention (such as pharmacologic, surgical, preventive, self-care)	Lines 72-73
	9b	Administration of therapeutic intervention (such as dosage, strength, duration)	N / A
	9c	Changes in therapeutic intervention (with rationale)	Lines 76-77
Follow-up and Outcomes	10a	Clinician and patient-assessed outcomes (if available)	Lines 82-87, 117-119
	10b	Important follow-up diagnostic and other test results	N / A
	10c	Intervention adherence and tolerability (How was this assessed?)	N / A
	10d	Adverse and unanticipated events	N / A
Discussion	11a	A scientific discussion of the strengths AND limitations associated with this case report	Lines 214-225
	11b	Discussion of the relevant medical literature **with references**.	Lines 187-200
	11c	The scientific rationale for any conclusions (including assessment of possible causes)	LInes 131-165
	11d	The primary "take-away" lessons of this case report (without references) in a one paragraph conclusion	Lines 211-213, 229-231
Patient Perspective	12	The patient should share their perspective in one to two paragraphs on the treatment(s) they received	N / A
Informed Consent	13	Did the patient give informed consent? Please provide if requested	Yes ☑ No ☐
